# Psychosocial effects of the corona pandemic on people with cancer: a qualitative study

**DOI:** 10.1007/s00432-023-04970-1

**Published:** 2023-06-20

**Authors:** Catalina Hoppe, Jens Büntzel, Kerstin Paradies, Marie Rösler, Jutta Hübner, Working Group Prevention and Integrative Oncology of the German Cancer Society

**Affiliations:** 1 Klinik für Innere Medizin II, Hämatologie und Internistische Onkologie, Universitätsklinikum Jena, Bachstraße 18, 07743 Jena, Germany; 2 Klinik für HNO-Erkrankungen, Kopf-Hals-Chirurgie, Interdisziplinäre Palliativstation Südharz Klinikum Nordhausen, Nordhausen, Germany; 3 Konferenz Onkologischer Kranken- und Kinderkrankenpflege, Deutschen Krebsgesellschaft e.V., Berlin, Germany; 4 Arbeitsgemeinschaft Soziale Arbeit in der Onkologie (ASO), Deutschen Krebsgesellschaft e.V., Berlin, Germany

**Keywords:** COVID-19 pandemic, Cancer patients, Psychosocial situation, Health care workers

## Abstract

**Introduction:**

The Corona pandemic caused far-reaching changes since 2020. We wanted to find out which factors determined the psycho-social well-being of cancer patients during the pandemic.

**Methods:**

From May to July 2021, structured interviews were conducted concerning lockdown, social limitations, the virus itself, the treatment situation, and opportunities.

**Results:**

Twenty people took part in the study (doctors, psychologists, nurses, social workers, patients). One of the most important aspects was the ban on visits. Others were the fear of infection and the possibility of vaccination. Wearing a mask seem to have been worse for the experts. Conflicts in families about the “right” behavior to protect yourself from infection have been a stressful issue for patients, just as the lack of balance and recreation in free time**.**

**Conclusion:**

Patients in the third wave of corona have become accustomed to the rules. Especially loneliness and the organization of time at home are psycho-social stress factors.

## Introduction

The outbreak of the Corona pandemic in 2020 brought with it far-reaching consequences. To counteract the spread, the government decided on rules of conduct and restrictions on contact with other people. This had a serious impact on both the processes in the clinics and the private sector. For patients, especially for patients with cancer, this resulted in restrictions and burdens which fundamentally affected cancer care. These included that relatives were no longer allowed to come along to doctor's appointments, visit patients in hospitals even in case of intensive treatments and, in the worst case, people died without accompaniment of loved ones (Büntzel et al. 2021a; Hübner 2020).

These new rules of behavior and the presence of the contagious virus affected not only processes and structures, but also the people's mental health. Surveys at three time points in April/May 2020, November/December 2020, and November/December 2021 showed that this affected both patients and the helpers, i.e., nurses, physicians, chaplains and psycho-oncologists (Büntzel et al. 2020, 2021a). It was noticeable that for a long time the helpers still perceived themselves as working normally and professionally, while patients perceived more and more the tense nature of the people and the situation as time went on. With increasing time, depressive moods were evident in 56% of professional helpers and in patients in 51% of cases (Hoppe et al. 2022).

In addition to the depressive symptoms, the fear of becoming infected, especially as a person with a history of cancer or under cancer treatment, brought with it new and existential fears. Even going to the doctor or clinic, the risk of infection was considered high (Büntzel et al. 2020). Consequences were a withdrawal from social life, friends and support groups. While few patients isolating at home even felt well and drew a secondary benefit not to expose to stressful social contacts as for example patients with head and neck cancer, others felt sheltered but longed for social contacts. With the opening of the lockdown, these patients on the one hand were eager to meet other people and on the other hand felt less safe than before. Moreover, after putting hope in the new vaccines, patients understood from the news that there were doubts on the effectiveness in case of cancer patients.

At the same time, first studies showed that patients estimated their own competences to prevent physical consequences of the pandemic as good. Yet, they reported a lack of strategies to be mentally prepared in and cope with the crisis (Büntzel et al. 2021b). For this reason, the aim of our study is to learn more which factors have a significant influence on the psycho-social well-being of cancer patients, one year after the onset of Corona?

## Method

### Study procedure

In a cross-disciplinary team consisting of representatives from medicine, psychology, nursing and social work, a model was developed, which included all factors that can have an influence on psycho-social well-being. First, the experts collected the different topics of society and interaction (Table 1, first column). Then, for each field, consequences to daily life were collected and differentiated (Table 1, second column). In this step the experts collected how these aspects of life were changed, presented new challenges or become unchanged or even better due to the situation with Corona (Table 1, third column). In a last step, the sequels for patients were collected (Table 1, fourth column). In the end, the model resulted in the following main aspects: working in lockdown, organizing everyday life, medical treatments, AHA rules (distance, hygiene, everyday mask, in German: Abstand, Hygiene, Alltagsmaske), media, free time in lockdown, the virus itself and digitalization (Table 1). The model was visualized in a mind map, which was consented on by the experts in a Delphi process.

**Table 1:** Basic ideas about the psycho-social effects of corona on cancer patients

Topic	General consequences	Consequences in more detail	Special consequences for patients
Work	Home office	New roles and routines	
		Less psychical activity	
		Change of daily structure	
	Home schooling	More responsibilities	
		Less time	Less time for own health care
	Time for family and activities		
Everyday life	Shopping		
	Appointments		
	Hairdresser, pedicure		
	Care of relatives		
	Use of public transport		
	craftsman		
	Administrative procedures		
Finances	Resources		
	Additional costs	Financial support for relatives due to job loss	Additional worry
Hospital treatment	Visitation ban	Loneliness in the hospital	Alone with stress, fears, processing, information
		Professional groups such as psychologists, pastoral care, Green Ladies…	
AHA rules	Masks	Worse understanding	
		Less empathy mediation	
		Greater distance	
	Restriction of social contacts	Less support	
		Less exchange	
		Less physical closeness	
		Limitations in self-help (first contact, participation in groups)	
		Intensification of existing relationships	
Media	Reports of numbers of dead and Infected people	Being at the mercy/helplessness	
	Measures, prospects, bans	Loss of control/inability to act	
Free time	Closed cinemas, theaters, Concert halls	Loss of free time/compensation	
	No vacation/traveling		
	Ban on groups	No self-help meetings	
	Closure of sports facilities	Fewer opportunities to move	
	Finding time in nature/with Yourself/peace		
	Essential reflection—lifestyle change	Improving diet and exercise	
The virus	People with Covid-19 in the hospital	Anxiety	Risk group with higher probability of complications
		Restrictions of visits	
Digitalization	Internet		
	Devices		
	Knowledge of web Search		
	Mailing		

### Interviews

We conducted semi-structured interviews via video call or telephone, which lasted a maximum of 30 min in total. In the course of this, representatives from the medical disciplines (doctors, psychologists, nursing staff and social workers) were asked open questions in five sections concerning lockdown (current challenges and consequences), social restrictions and rules (interactions with mask), the corona virus in general (new fears), the treatment situation and chances of corona for cancer patients. The interview for the patients also included open questions in different five parts. The last part was an open one, the other thematically equal to the interviews of the experts. The only difference was that the data were self-assessments.

The interviews were conducted over a period of four months—from March 2021 to July 2021.

### Recruitment and participants

We asked the working groups within the German cancer society (AG prevention and integrative medicine in oncology) and the independent DAPO (German working group for psycho-social oncology) for contact persons for the expert interviews. Patients were addressed by physicians (JH) or via self-help groups. Before participating, an information sheet and a declaration of consent were sent by email and returned by the participants. Recruitment continued until theoretical saturation (i.e., no new emerging themes) was reached.

Included participants were (a) doctors, (b) psychologists, (c) nurses, (d) social workers and (e) patients who with cancer during or after treatment.

### Analysis of the data

The interviews were audio-recorded and transcribed. Based on the model created at the beginning, the content was structured using the model and analyzed. In this way, five sub-models were created from the various responses from experts and patients, which summarize the psycho-social effects of the corona pandemic for people with cancer. These can be compared with the initial model and with each other. It was important to note that not all groups of participants always gave responses to every aspect of the model.

## Results

A total of 3 doctors, 5 psychologists, 4 nurses, 4 social workers and 4 patients were interviewed. 80% of these 20 respondents were female and 20% male (among the patients the distribution was 2 and 2).

### Doctors

In their interviews, the doctors reported the *restrictions of social contacts* meant a high burden for families where a member was affected by cancer. Family members wanted to keep distance in order not to infect the patient and at the same time wanted to keep contact and provide support (Table 2, nr. 1). Wearing *masks* on the one hand was accepted as necessary protection. In the other hand, non-verbal communication by mimics, especially offering support by smiling was stopped. Moreover, having to speak louder also made a less friendly atmosphere (Table 2, nr. 2). On the other hand, it was a matter of getting used to and becoming normal. Some physicians said they paid more attention to the posture to compensate for the missing facial expressions (Table 2, nr. 3). Another important aspect the doctors brought in related to the *corona virus* itself or the fear of infection. It was found that people kept coming to the hospital but there were fewer initial diagnoses and patients were doing more information search from the internet. The requests for rehab are large, but fewer patients actually went to rehab clinics, which in part could have been due to the fact that official matters were more difficult to organize online (Table 2, nr. 4). Overall, from the doctors' point of view, the situation in the clinics has been safe since testing had been established and cancer patients had strictly adhered to the rules and mostly behaved in an exemplary manner. The possibility of vaccination emerged as a new topic towards the middle of the year, which resulted in new questions from patients. On the one hand, they were interested in how to get their turn and on the other hand, they worried not getting the protection early enough (Table 2, nr. 5). In terms of patients' *financial situation*, doctors saw no differences due to Corona (Table 2, nr.6).

**Table 2:** Quotations from the interviews with the doctors

Number	Quotation
1	‘(…) and also, within families, because of course everyone is considerate and keeps their distance, it's such a balancing act, so you want contact, but on the other hand you don't want to endanger anyone and still have this great need for contact.’
2	‘I find that the louder you get, the more energetic your voice becomes and not softer and friendlier.’
3	‘You just don't see each other that directly, sometimes the facial expressions aren't judged so well, you're more dependent on your posture (…), also in the assessment, but that's a mutual problem (…) so I didn`t notice that it had any major impact.’
4	‘Especially for patients who are not intellectually very fit, who tend to have a lower level of education, who already have difficulties with the authorities and are then supposed to do the whole thing online or by telephone, they will certainly find it even more difficult than under normal people conditions.’
5	‘At the moment the questions about vaccine doses for the patients – for those affected – are in the foreground. (…) How do I get an extra certificate, do I belong to the risk group, does my cancer belong to the situations with a higher risk or is it to be assessed as rather lower.’
6	‘No, the rehabilitation is fully financed, so it doesn't really matter.’

### Psychologists

The ban on visits were consistently rated negatively by the psychologists. They described suffering, feeling of being alone, a lack of resources, a lot of personal responsibility and also suffering on the part of the relatives (Table 3, nr. 1). The general *restriction of social contacts* was also assessed as consistently negative as titled to loneliness, loss of social togetherness, physical contact and practical support and the emergence of new conflicts about views regarding the rules (Table 3, nr. 2). Younger patients would have been able to compensate for some of the loneliness through *digital opportunities*. However, at the same time, a digital surfeit was noticeable among patients and online offers had become less attractive (Table 3, nr. 3). When it came to *masking*, there was a minority with the opinion that they would not restrict communication with the patients. Yet, many psychologists said that it would make work more difficult in the group settings because it was less easy to evaluate mood of the patients and more difficult to convey empathy (Table 3, nr. 4 and 5). In addition, it is more difficult to recognize other persons with whom one is not very familiar and in everyday hospital life this was exhausting. The fear of infection with the *corona virus* did not fail to appear in the stories of psychologists regarding new stressors for the patients. It would lead to withdrawal, strict obeying of rules, and rejection of help at home. On the other hand, many patients would still like to use the rehab offer and were looking forward to the social contacts there. The safety provided by tests and rules was rated as helpful, stated the psychologists. Moreover, patients were reported as generally less burdened by the virus as they had already dealt with the issues of death and dying before the pandemic. Accordingly, these fears were not completely new for them (Table 3, nr. 6). Regarding *everyday life*, the psychologists estimated the uncertainty for patients when going home to be greater than in the past (Table 3, nr. 7) and reported that patients anticipated more conflicts in families due to the question of how strictly to implement the rules. In the patients` *free time* there were less or no opportunities for distraction, doing something good for oneself and for one’s own balance (Table 3, nr. 8). With the *home office*, patients would not have less time for themselves, but on the other side would also be alone less often (Table 3, nr. 9). Home Schooling might be a double burden, especially for parents affected by cancer. Moreover, and looking after children might in some cases even be a reason why parents would not go to rehabilitation clinics (Table 3, nr. 10). *Financially*, little has changed, according to the psychologists. The patients were on sick leave and in case of independence before the illness, it had always been a major financial problem (Table 3, nr. 11).

**Table 3:** Quotations from the interviews with the psychologists

Number	Quotation
1	‘And then, um, of course there are also the people, who, um, who are very much on their own when they go to the therapists – that is, when they go to the hospital or to the doctor's office or something. Nobody is allowed to come along, nobody is allowed to come along to talks – and they are then very alone, more than before, when people often went with them or picked them up or who were allowed to come to the talks or – if they were in the hospital, no one at all is allowed to have visitors or the relatives who suffer a lot because they are not allowed to visit them.’
2	‘What she had in terms of social contacts has actually been reduced to phone calls (…). And then there were quarrels among friends because they have different opinions, so we see that everywhere, that in the families, with the patients, with the teams, with everyone, that there are different opinions about what is justified, what is not justified, how should we deal with it and it actually led to her – very extreme for her – but it actually led to a suicidal crisis for her.’
3	‘Of course, that's (digital yoga offers) not the same, but in the beginning it was maybe a good bridging, but meanwhile also such a fatigue.’
4	‘I find it extremely problematic. At some point I had an illness coping group that was very large, I couldn't even see their eyes properly. I found that, so I found it really terrible, because then you can't cope with the disease in a reasonable way.’
5	‘At first the patients said that the greeting culture had changed so much. Staff wouldn't say hello like that anymore, but then somehow it came out that a smile when you walk through the house is no longer seen, so you have to nod more clearly or say something louder to prevent understanding.’
6	‘Well, I found it very interesting that death and dying is being carried into society on a massive scale by the media and that of course those who haven't dealt with it at all are really flashed to the point that they got panic attacks. And some of the cancer patients actually dealt with it more calmly. Even though they are high-risk patients.’
7	‘What's also on top of that is how can I get all these Corona rules right now, when I might be at home between therapies. How can I master this? That wasn't the issue before. It was a pleasure to be able to go home.’
8	‘Actually, as an additional burden. So don't be distracted. To be able to brood a lot. Having nothing to really look forward to.’
9	‘People who were used to having time to themselves at home no longer have it because who knows what's going on in the home office. But there are also others who say, that's great, there's always someone at home now. So, there's both.’
10	‘(…) that parents who were ill then felt more like they were being condemned or responsible for guiding the homeschooling when the other spouse had to work, yes and then, you're at home, then you don't go to rehab because of the children. And that perhaps the use of rehab by parents has simply decreased because they are needed and even if they can only lie on the sofa, that is care, which of course is also very tiring.’
11	‘So, business and cancer and finance and cancer and money, it's a current topic anyway. I haven't experienced any noticeable differences since Corona.’

### Nurses

As a consequence of the *ban on visiting* in clinics, the nursing staff also reported isolation and a lack of contact to family members and other caregivers. They also found themselves to be in charge of replacing this loss (Table 4, nr. 1). Some patients would be understanding in terms of loneliness in clinics, others angry. Others would be overwhelmed and others resigned in that they would have to make decisions for themselves anyway. Like the doctors, the nurses also reported that exceptions to the ban on visits were made in the palliative care and also for some consultations (Table 4, nr. 2). As a positive consequence of the restrictions, they reported the absence of "secondary" patients in the form of relatives. In part, especially for younger patients, the absence of relatives can be compensated for by *media*, caregivers said. However, others also described having no WLAN reception on the station and thus not being able to make offers at all. The attitudes towards the *mask requirement* were heterogeneous also. The lack of facial expressions would make it harder to understand nonverbals. Yet, they asserted that practice would make perfect. The threshold to communicate reportedly was higher as it was harder to understand each other and the distance was greater (Table 4, nr. 3). On the other hand, the mask had become very accepted and commonplace. For most of the patients it would mean safety. Moreover, a mask might also provide a way to hide themselves (Table 4, nr. 4). However, nurses also reported that the mask led to the patient eating and drinking less in day clinics, which would make them feel worse back at home. Concerning management of side effects, it was rated as important to look under the mask to assess side effects in the region of the head and to prevent side effects for example on the oral mucosa which might lead to discontinuing or reducing of the treatment dose (Table 4, nr. 5). With regard to the *corona virus*, the nursing staff said the same as the doctors. The fear of infection was high, but the clinics paid attention to compliance with the rules and distances (Table 4, nr. 6). At home, however, patients often refused help out of fear which made outpatient care more difficult. As the experts already reported, also the nurses did not notice differences in the financial situation for people with cancer since Corona. However, they added that concerns about returning to work had increased (Table 4, nr. 7).

**Table 4:** Quotations from the interviews with the nurses

Number	Quotation
1	‘So, the problems and missing relatives were much bigger and especially for the patients who were diagnosed for the first time (…), it was a problem because the relatives were not there.’
2	‘In our clinic, there is still a ban on visiting patients, except for pre-final patients – i.e., palliative patients who are in the process of dying, exceptions are made there, but normal oncological patients are not entitled to visits. And that is very, very stressful, especially when it comes to decisions about any new therapies or operations or whatever.’
3	‘This simply increases the inhibition threshold to communicate. The patients don't talk about their problems as much anymore, or if you ask them something, they don't really understand it, because you speak a little more subduedly.’
4	‘A patient once said to me whether the thing is annoying or not, this is actually about my own protection. She literally said that to me once: whether I like it or not, I'd rather put it on because I don't want to catch anything.’
5	‘So, I wouldn't say it was days late or that patients didn't report something because they knew relatively well what it was, you have to get used to asking specific or different questions and it took a moment. It was more difficult at the beginning and it wasn't that present back then, but it's now become part of everyday life.’
6	‘At the beginning, i.e., last year during the first lockdown, fewer patients came because there were also fears of going to the hospital at all.’
7	‘(…) younger patients, for example, who are on short-time work or who have become unemployed due to the pandemic, naturally have a much greater problem of existence due to a tumor disease, because they say that they will no longer be able to find work or that they will not be rehired after short-time work.’

### Social workers

The *ban on visits* led to loneliness of the patients and thereby increased fears and was a reason why some did not want to go to the hospital. With longer stays, the patients’ resignation was increasing, but at the same time the feeling of misfortune, the withdrawal from social contacts and also the aggressiveness (Table 5, nr. 1). All in all, the current situation also meant that seriously ill people staid longer in hospitals than in a palliative care unit or in a hospice as the latter reduced their capacities. Considering the patient's point of view, the social workers saw the *mask requirement* as a possibility of protection and an often accepted method (Table 5, nr. 2). on the one hand. For patients with difficulties of breathing wearing a mask also was seen as a heavy burden (Table 5, nr. 3). For the specialists themselves, it was more exhausting during their work and would require more attention to be able to be aware of changes in the patients’ status solely from the eyes. In the *everyday life* of patients, Corona had resulted in less support at home (Table 5, nr. 4). Voluntary helpers were not able to come, housekeeping, as well as care services and homecarers were rejected often in fear of infection. New conflicts arose and it was particularly difficult for children of cancer patients to maintain normality in everyday life. The lockdown meant that sick people sometimes were better cared for when members of the family were able to stay at home. On the other hand, it is an additional burden for cancer patients to look after their children at home in situations in which they were hardly able to cope with stress (Table 5, nr. 5). The restrictions on social contacts in the private sector meant that the patients would have less relief, became lonelier and received less closeness and comfort. For some individuals, however, it also meant a legitimation to withdraw. The restrictions in *leisure time* meant, on the one hand, that fewer resources were available and the recovery time was more difficult (Table 5, nr. 6). Over the time with Corona, many became shy and step outside were often difficult (Table 5, nr. 7).

**Table 5:** Quotations from the interviews with the social workers

Number	Quotation
1	‘On the other hand, you notice that especially the second and third time, when the second or third stay in hospital takes place, it is much harder. The unhappiness is greater, the loneliness is greater, the withdrawal into oneself is greater.’
2	‘Well, most of them really kept to the requirements and thought it was good and right and did it that way, especially when they were also undergoing chemotherapy, of course they also felt protected by it.’
3	‘If the overall weakness is relatively high, then down to the X-ray, to the CT—that is incredibly exhausting for the patient.’
4	‘So, there is a great deal of caution and therefore a stronger sense of being left alone. (Who otherwise might have taken the grandchildren and provided relief)’
5	‘Single mother with two school-age children who are homeschooling and the mother is undergoing chemotherapy. This is the super meltdown. So, the two growing children in the home office at home and you yourself are not resilient at all right now.’
6	‘There were activities that would otherwise have been carried out regularly, but then they were no longer possible. So, sporting activities, but also social contacts and that of course also made the time of recovery very difficult. There are still spaces to be opened up and to see what remains, what cannot be sold despite Corona. (…) It really was always a search.’
7	‘Because the younger ones, yes, they were just happy that meeting is now possible and yes, they did not find it that difficult, but older people, who then made it up like that, in this little world (…) they are really shy to go out again.’

### Patients

In the reports of patients, it became clear that the *restriction of social contacts* was perceived as stressful because meeting their family was no longer possible. There was hardly any contact with other people. Moreover, conflicts arose as the patients reported to have to justify their behavior and withdrawal from contacts (Table 6, nr. 1). However, there were also statements that the restrictions were not perceived as being too burdensome. In addition to private contacts, the self-help groups were also affected. Some members of groups reported that they had not seen each other for two years and others who had switched to online meetings and were able to continue to talk to each other regularly (Table 6, nr. 2, 3). The subject of *digitization* was perceived very differently by the patients. Some said that in their support groups there was a lot of rejection and fears. Others said that Corona gave them access to the digital world. They joined online groups, were able to take part in events taking place across Germany and learned a lot of new things (Table 6, nr. 4). The *mask requirement* was not experienced as restrictive. In their statements, the patients made clear that they were already used to it and that the masks would convey a sense of safety (Table 6, nr. 5). Communication also worked by eyes. A big topic on the part of the patients was the fear of infection with the *corona virus*. They were very aware that they have worse prospects in case of an infection, so they stack to the rules and withdraw into their own homes (Table 6, nr. 6). Sometimes it also made the patients feel uncomfortable in full doctor's offices. Moreover, they chose not to take part in rehabilitation and physiotherapy. Vaccination was also new and important for many patients. It brought around hope and confidence that better times would come (Table 6, nr. 7). A lot of effort was made to receive the vaccination. At the same time, this gave rise to new uncertainties and it was stated that tumor therapy still had priority and was more important. When asked how patients would perceive their *free time* in times of pandemic, the answers were heterogeneous. They reported that they were not able to track their physical activities such as dancing and swimming, as well as traveling and vacationing (Table 6, nr. 8). On the other hand, it was said that the main restrictions in everyday life and leisure time came from the cancer disease itself and not from Corona (Table 6, nr. 9). The restrictions are tolerable, they were still able to maintain quality of life and, for example, find joy in things at home. The relativization was easier because for them, the most important thing is to live. Modesty was also higher. A final issue that came up with patients was the role of the *public and the media* during the pandemic. The ever-increasing incidence has led to a spiral of worries and little consolation and perspective have been given, but rather fear and a lack of hope.

**Table 6:** Quotations from the interviews with the patients

Number	Quotation
1	‘There are certain people who think differently and then you have to justify yourself. And explain why you do not do this and that and that's for me (…) I also avoid contact with it; I do not want anything to do with it. I also shut down my entire private environment there.’
2	‘It's also quite difficult at the moment, because there are also older people, so many are much older than me and there are still some who are afraid now, so some of them do not go out at all anymore (…). We normally had such a meeting in Lüneburg once a month. But the meetings are currently—everything has been canceled—that's almost two years now. Two years that we could not do any more meetings there.’
3	‘Once a month it always took place with personal meetings and since Corona it has all broken down because it is also on a hospital site and we were basically not allowed in there either and we had started doing it online once a month.’
4	‘For me, that was really my entry into the online world (…) I really went through a learning process that I would not have done without the Corona. Because then you would have visited everything on site, so you would not have been able to visit some of the event at all, because they are also very far away.’
5	‘But I've gotten used to it, so it does not bother me at all anymore. And I'm there too, it's on the safe side for me too, I wear them because that's the way it's supposed to be.’
6	‘So, if you're pretty affected and are afraid yourself that if you get caught by the virus and that you'll die, then you're looking for a certain security.’
7	‘And when you then, when you now hear the numbers, when you hear the numbers—20%, 23%, 24%, 30%—so I believe that this will also very slowly improve the basic mood of some people.’
8	‘Visiting museums is not an option, meeting friends is not an option—of course I'm still active in music, and none of that either. I can neither sing nor meet my friends to make music. There's a lot that does not work.’
9	‘As a result, I have extreme mobility problems, among other things, but that did not come from COVID or has nothing to do with COVID at all. It actually came from cancer therapy. Or my reaction to it.’

## Discussion

The results of the interviews show that the psycho-social effects of the corona pandemic are far-reaching in the third wave of the pandemic in Germany for cancer patients. It is noticeable that the assessments are all very heterogeneous and are mostly seen from a critical and a positive side. In all occupational groups it is clear that the ban on visits plays one of the most important roles and has the most serious consequences suffering, loneliness, being left alone with decisions and a lack of resources. Followed by the consequence of the fear of infection with the corona virus as a patient who is already at risk. The obligation to wear masks entails a need for change and new challenges, but has become more normal over time or can be compensated for. New topics added in the summer of last year were in particular the possibility of vaccination and the associated uncertainties with regard to tumor therapy.

Depending on their fields of activity, the doctors and nursing staff tended to describe the situation in the hospitals, with the psychologists and social workers also mentioning the situation at home with work, children and leisure time. In their reports, it also became clear that new potential for conflict due to the corona rules in families and greater insecurity at home with less external help are also consequences of the pandemic. At home there is a double burden and there is less balance through leisure activities, which primarily affects recovery after the illness. The patients brought additional insights into the situation with self-help groups and the helper system connected with it, which has been severely affected by the pandemic.

Compared to our starting basic assumptions about the psycho-social effects of Corona on cancer patients we can state the following. In terms of work, it was mentioned much less frequently—especially by the patients—and therefore seems to play a smaller role than assumed. For parents, the effect that this means they can take less care of their own health seems to be true. A new aspect was that returning to work had become more difficult.

The challenges of everyday life like shopping, appointments or use of public transport were also not given serious importance. According to the results of the interviews, it was not a factor that altered or influenced the psycho-social well-being of cancer patients during the pandemic. However, it could well be that many of these aspects were supported and made feasible by relatives. There are indications from other studies that say that partners of cancer patients are particularly affected by the consequences of the pandemic due to the lack of offers of support (Büntzel et al. 2021a).

The aspect of loneliness during the treatment in the clinic could be strongly reinforced by the statements of the professionals and the patients. Overall, it seems to be one of the most important aspects that significantly changed the psycho-social well-being of the patients in a negative way. This fact is also confirmed by studies from previous surveys (Büntzel et al. 2021b). The problem seems to have been present since the beginning of the pandemic and has not yet found an adequate way of dealing with it in the third wave.

Regarding the AHA rules, it ultimately became apparent that we had primarily considered negative aspects in advance. These were also listed by the interviewees. Nevertheless, it turned out at the same time that, for many the mask requirement was normal in advance or during the time of the pandemic. At the same time, protection is appreciated. What has become clear both from the statements of the experts and from the patients is that new issues have arisen in families concerning appropriate behavior and justifications for certain behaviors. This could be one of the factors contributing to the fact that the distress level was always high in many of the patients, even in other studies (Edge et al. 2021). A study from Italy also showed that cancer patients with stable family networks had less psycho-social impact of the pandemic (Ferrara et al. 2021).

We estimated the role of the media to be greater. it does not seem to have been the focus of attention as an influence but it has been said among patients that it has contributed to worry and hopelessness. Here, a certain habituation seems to have taken place over time, as in the first wave, surveys still showed that 71% of the patients were very or moderately irritated by the information distributed by the media (Büntzel et al. 2020).

With regard to free time, there were only a few comments, but they made clear that as a consequence of the restrictions in this area, above all the lack of compensation, resources and opportunities for exercise play a role. However, the patients also reported that the greatest limitations would come about as a result of the disease which would indicate that Corona is only a small additional factor here. In the end, it is probably a combination of both aspects—the disease and the restrictions imposed by the COVID regulations. Schmidt et al. (2021) showed in this regard that physical activity decreased significantly in all age groups during the first wave and explained this fact mainly with the inaccessibility of gyms and the suspension of professionally supervised training, such as rehabilitation sports.

The virus itself actually played a major role and had a strong impact on psycho-social well-being in that it increased anxiety. A fact that Butow et al. (2022) found in their study a year earlier. What has changed since then is that many tried to take away this uncertainty by getting vaccinated.

## Limitations

The study has some limits, which are briefly discussed below. The qualitative survey methods offer opportunities to record individual factors and to go in depth with the individual respondents. At the same time, their results can only be generalized to a limited extent. In addition, the sample size is relatively small which is why it is unclear, especially in the patient subsample, whether information saturation was reached. Quantitative investigations with a larger sample from the knowledge gained here on influences on the psycho-social condition of the patients should follow to be able to make generalized statements.

## Conclusion

Previous studies of changes during the pandemic have focused on the care situation and the impact on psycho-social well-being. We therefore wanted to focus on the social context and see what makes the differences here.

Aspects such as work and normal daily activities as well as the role of the media seem to influence psycho-social well-being less than perhaps had been assumed. Patients in the third wave have also, it seems, already become accustomed to the AHA rules. The two most serious and stressful aspects in the third wave still seem to be the loneliness caused by the visiting ban and the possible infection with the Corona virus. All in all, the results suggest that the period after the illness with renewed exercise and the return to work is also affected by the pandemic and making it harder for patients than it was before the advent of COVID-19 and its consequences. Another finding is that on the one hand, relatives seem to buffer some of the stress, and on the other hand, new disagreements arise when it comes to the right behavior during the pandemic, which on the one hand relieves the oncological patients, but on the other hand also exposes them to additional discussions.

## Data Availability

The data that support the findings of this study are not publicly available due to their containing information that could compromise the privacy of research participants but are available from the corresponding author CH upon reasonable request.
